# Anatomical Augmentation Using Suture Tape for Acute Syndesmotic Injury in Maisonneuve Fracture: A Case Report

**DOI:** 10.3390/medicina59040652

**Published:** 2023-03-25

**Authors:** Sung-Joon Yoon, Ki-Jin Jung, Yong-Cheol Hong, Eui-Dong Yeo, Hong-Seop Lee, Sung-Hun Won, Byung-Ryul Lee, Jae-Young Ji, Dhong-Won Lee, Woo-Jong Kim

**Affiliations:** 1 Department of Orthopaedic Surgery, Soonchunhyang University Hospital Cheonan, 31, Suncheonhyang 6-gil, Dongam-gu, Cheonan 31151, Republic of Korea; 2 Department of Orthopaedic Surgery, Veterans Health Service Medical Center, Seoul 05368, Republic of Korea; 3 Department of Foot and Ankle Surgery, Nowon Eulji Medical Center, Eulji University, 68, Hangeulbiseok-ro, Nowon-gu, Seoul 01830, Republic of Korea; 4 Department of Orthopaedic Surgery, Soonchunhyang University Hospital Seoul, 59, Daesagwan-ro, Yongsan-gu, Seoul 04401, Republic of Korea; 5 Department of Anesthesiology and Pain Medicine, Soonchunhyang University Hospital Cheonan, 31, Suncheonhyang 6-gil, Dongam-gu, Cheonan 31151, Republic of Korea; 6 Department of Orthopaedic Surgery, Konkuk University Medical Center, 120-1, Neungdong-ro, Gwangjin-gu, Seoul 05030, Republic of Korea

**Keywords:** syndesmosis injury, instability, suture tape, anatomic augmentation

## Abstract

Ankle syndesmosis is crucial to the integrity of the ankle joint and weight-bearing; an injury to this structure can lead to significant disability. The treatment methods for distal syndesmosis injuries are controversial. The representative treatment methods include transsyndesmotic screw fixation and suture-button fixation, and good results with suture tape augmentation have recently been reported. However, an augmentation using suture tape is only possible when the posterior inferior tibiofibular ligament (PITFL) is intact. This study describes the case of an unstable syndesmosis injury, accompanied by anterior inferior tibiofibular ligament (AITFL) and PITFL injuries, which were treated successfully using suture tape. A 39-year-old male patient sustained right ankle damage while skateboarding. His leg and ankle radiographs revealed a widening of the medial clear space, a posterior malleolus fracture, a reduced “syndesmosis overlap” compared with the contralateral side, and a proximal fibula fracture. The magnetic resonance imaging revealed ruptured deltoid ligaments, accompanied by AITFL, PITFL, and interosseous ligament injuries. A diagnosis of a Maisonneuve fracture with an unstable syndesmotic injury was made. The patient underwent an open syndesmotic joint reduction, along with an AITFL and PITFL augmentation. This anatomical reduction was confirmed using intraoperative arthroscopy and postoperative computed tomography (CT). An axial CT that was performed at the 6-month follow-up exam revealed a similar alignment of the syndesmosis between the injured and uninjured sides. There were no surgical complications and the patient did not complain of discomfort in his daily life. At the 12-month follow-up exam, a good clinical outcome was confirmed. As a treatment for unstable syndesmosis injury, ligament augmentation using suture tape shows satisfactory clinical outcomes and can be considered as a useful and reliable method for anatomical restoration and rapid rehabilitation.

## 1. Introduction

The tibiofibular syndesmosis, a fibrous joint that stabilizes the fibula and tibia, consists of four lateral ligaments: the anterior inferior tibiofibular ligament (AITFL), interosseous ligament (IOL), transverse ligament (TL), and posterior inferior tibiofibular ligament (PITFL). These ligaments stabilize the syndesmosis and prevent the excessive motion of the fibula, such that an appropriate fibular position is maintained; they also play an important role in syndesmotic function and the talar position [1]. Within the syndesmotic ligament complex, the AITFL and PITFL play the most important roles in stabilizing the distal syndesmosis [2].

Distal tibiofibular syndesmotic injury is involved in 10% of all ankle fractures and up to 20% of rotational ankle fractures [3,4,5]. The distal tibiofibular syndesmosis is crucial for the congruity and integrity of the ankle joint, which, in turn, is critical for weight-bearing [6,7]. An injury to these critical structures can lead to significant disability [8,9,10]. According to a cadaveric study [11], in cases of syndesmosis injury, tibiotalar contact pressure can be reduced by 42% with only a 1 mm lateral shift of the talus. The stabilization of the syndesmosis is essential to achieving good long-term, functional outcomes for the ankle joint, and to preventing posttraumatic arthritis [5,12].

One traditional method for reducing the syndesmosis is a transosseous screw fixation. However, the position, diameter, number, and retrieval of the syndesmotic screws, as well as the method of cortical fixation, remain controversial [13,14,15,16]. Recently, several studies have reported the use of suture tape for a ligament augmentation in cases of syndesmosis injury [17,18,19,20]. In one study, this novel fixation method proved to be as effective as screw fixation [21], while, in a cadaver model, a minimally invasive anatomic augmentation of the anterior and posterior syndesmosis was achieved by using suture tape [22]. In this study, we report a case of unstable syndesmotic injury, in which the anatomical reduction of the syndesmosis was achieved by an augmentation of the AITFL and PITFL using suture tape.

## 2. Case Presentation

This case report was approved by the Institutional Review Board (IRB) of Soonchunhyang University Cheonan Hospital, Cheonan, South Korea (IRB No. 2023-01-007). The patient provided written informed consent for the publication of this report and the accompanying images.

A 39-year-old male presented to the emergency department of our hospital with severe pain and swelling in the right ankle. The patient stated that he fell off a skateboard and rotated his ankle. He had no history of illness, or of genetic or familial diseases. A physical examination revealed ankle swelling, extreme tenderness, and ecchymosis in the medial aspect of the ankle and the proximal fibula. There were no neurological deficits, and the dorsalis pedis and tibialis posterior arteries were palpable.

**Figure 1:**
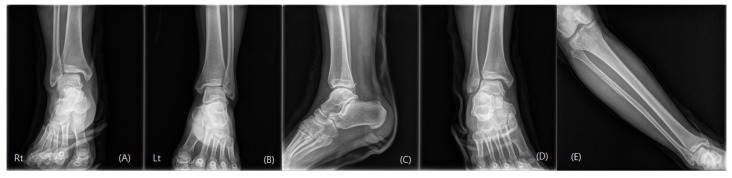
Preoperative plain radiographs showing widening of the medial clear space ((**A**) anteroposterior view of the site of injury, and (**B**) anteroposterior view), a posterior malleolar fracture ((**C**) lateral view), reduced syndesmosis overlap ((**D**) mortise view), and a proximal fibula fracture ((**E**) full-length radiograph of the lower leg; anteroposterior view).

**Figure 2:**
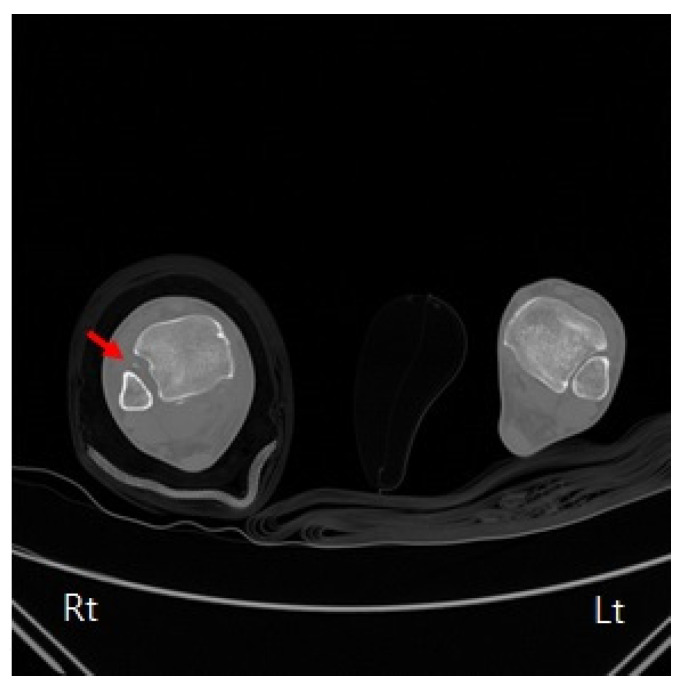
Preoperative axial computed tomography scan showing a syndesmotic injury in the right ankle. It can be seen that the fibula is dislocated from the fibula notch (red arrow).

**Figure 3:**
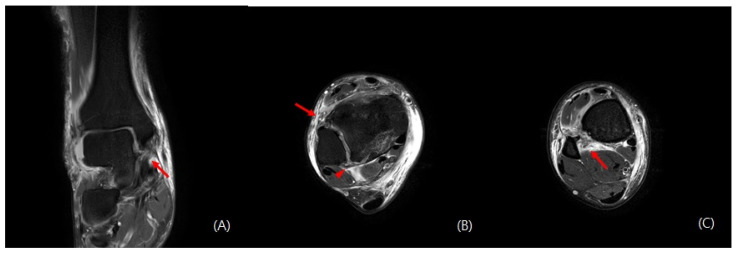
Coronal magnetic resonance imaging (MRI). An area of high signal intensity (red arrow) indicates a deltoid ligament injury (**A**). Axial MRI showing the anterior inferior tibiofibular ligament (red arrow) and posterior inferior tibiofibular ligament (red arrowhead) injuries (**B**). Axial MRI showing interosseous membrane rupture (red arrow) (**C**).

**Figure 4:**
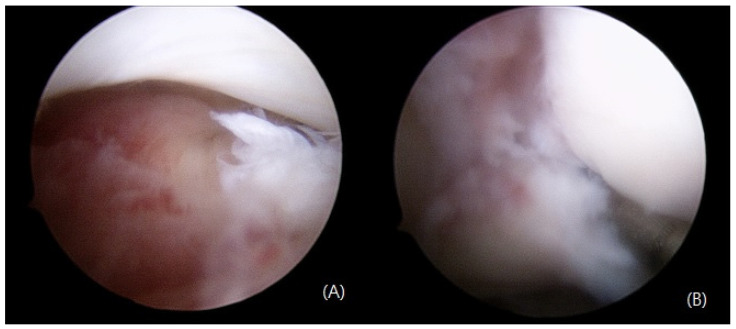
Intraoperative arthroscopic findings of syndesmotic injury with widening of the gap between the fibula and tibia (**A**), and posterior inferior tibiofibular ligament rupture at the point of tibial insertion (**B**).

**Figure 5:**
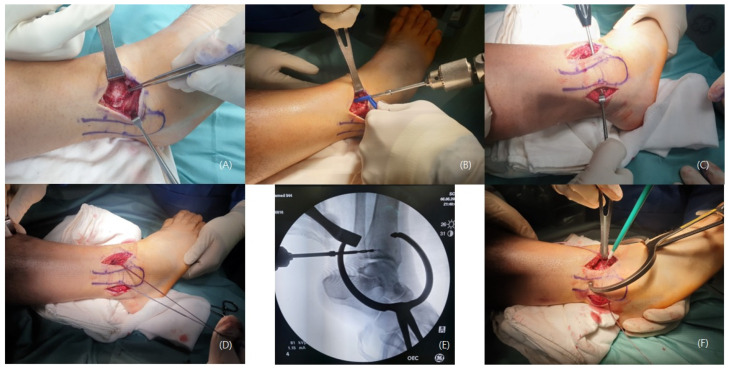
(**A**) Confirmation of anterior inferior tibiofibular ligament rupture. (**B**) Check of the distal tibial footprints and a 3.4 mm bone tunnel was created. (**C**) In the syndesmosis ligament in the distal fibula using a 2.7 mm drill. (**C**) A 2.7 mm drilling was performed on the footprints of the syndesmosis ligament in the distal fibula from front to back to create a bone tunnel. (**D**) Suture tape was passed through the bone tunnel and fixed with interference screws. (**E**) Under C-arm guidance, a bone tunnel was created in the posterior syndesmosis on the Volkmann tubercle side. (**F**) After reducing the syndesmosis joint, the free ends of the suture tape were fixed with 4.75 mm SwiveLock screws.

**Figure 6:**
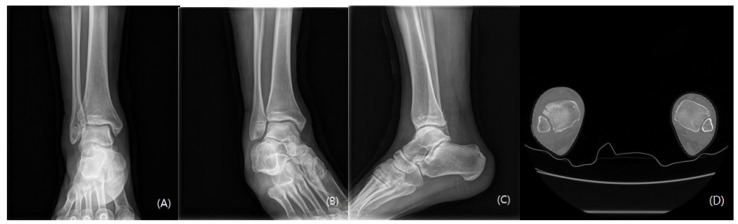
The syndesmosis joint was clearly reduced on the postoperative X-ray ((**A**) anteroposterior view, (**B**) lateral view, and (**C**) mortise view); this was confirmed by axial computed tomography (**D**).

The anteroposterior, lateral, and mortise view right ankle radiographs revealed a widening of the medial clear space and a posterior malleolus fracture. Moreover, the “syndesmosis overlap” was reduced in comparison with the contralateral side. Additionally, a full-length radiograph of the lower leg revealed a proximal fibula fracture (Figure 1). Computed tomography (CT) scans were taken for an accurate evaluation of the syndesmosis. On the axial CT, the fibula was not located in the fibula notch; it was found to be displaced laterally and posteriorly at a point 1 cm above the tibial plafond (Figure 2). The magnetic resonance imaging (MRI) revealed that there were ruptured deltoid ligaments, along with AITFL, PITFL, and interosseous membrane (IOM) injuries (Figure 3). The final diagnosis was a Maisonneuve fracture with a proximal fibular fracture, a syndesmosis injury with an IOM rupture, and a medial deltoid ligament injury; these findings were confirmed during surgery. On day 2 after the injury, the patient underwent a syndesmosis reduction and fixation. The patient was placed on the operating table in the supine position, and arthroscopy was performed using standard anteromedial and anterolateral portals. We did not observe a cartilage injury, syndesmotic instability (lateral malleolus displacement >5 mm), or PITFL rupture at the point of the tibia insertion (Figure 4). We planned to use suture tape for the syndesmosis joint reduction and fixation. InternalBrace (Arthrex, Naples, FL, USA), a nonabsorbable suture tape, was used for the fixation. First, the AITFL rupture was confirmed to be approximately 4 cm above the distal tibiofibular joint. We checked the distal tibial footprints and a 3.4 mm bone tunnel was created. A 2.7 mm drilling was performed on the footprints of the syndesmosis ligament in the distal fibula, from front to back, to create a bone tunnel. The suture tape was passed through and fixed with 3.5 mm interference screws (SwiveLock; Arthrex). After internally rotating the patient’s leg, a longitudinal incision was made approximately 5 cm above the Volkmann tubercle. We palpated the Volkmann tubercle and passed the suture tape between the peroneus tendon and the bone. After reducing the syndesmosis joint, the free ends of the suture tape were fixed to the bone tunnel on the tibia side, which was prepared under C-arm guidance with 4.75 mm SwiveLock® anchors (Figure 5). Then, the medial clear space was reduced to within the normal range. A deltoid ligament repair was not performed and the proximal fibula fracture was treated conservatively. A plain X-ray and CT were performed immediately after the surgery had confirmed a successful syndesmotic reduction (Figure 6).

**Figure 7:**
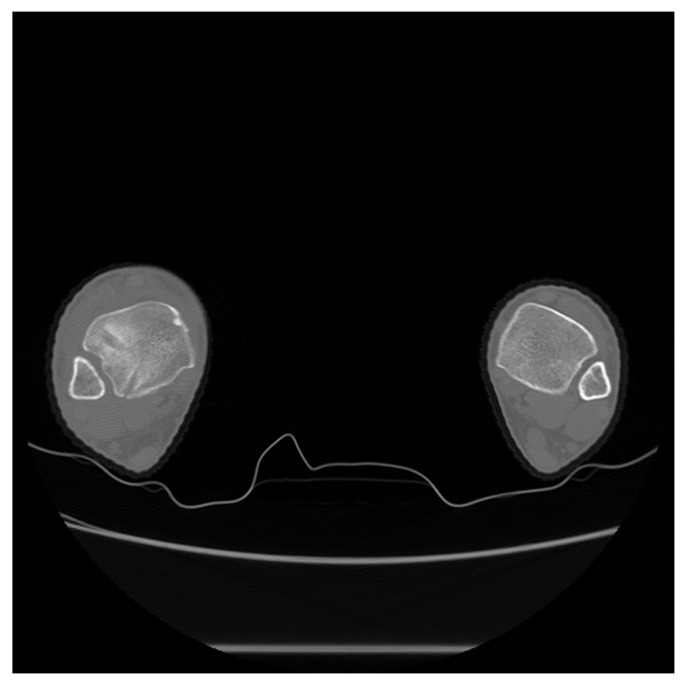
Axial computed tomography performed at the 6-month follow-up exam revealed similar alignment of the syndesmosis between the injured and uninjured sides.

Postoperatively, a short leg splint was worn for approximately 2 weeks. The patient was instructed to use an ankle brace for an additional 2 weeks. Active and passive ankle range of motion exercises were performed from 4 weeks postoperatively, and full weight-bearing walking was then allowed with braces. The braces were removed after 6 weeks. Then, a 3-month rehabilitation program consisting of ankle muscle strength, balance, and functional performance training was completed. An axial CT that was performed 6 months after the surgery revealed a similar alignment of the syndesmosis between the injured and uninjured sides (Figure 7). There were no complications and the patient did not complain of discomfort in daily life.

At the 1-year postoperative follow-up exam, the Olerud–Molander Ankle Score and The American Orthodefic Foot and Ankle Society Ankle-Hindfoot scale were at 95 and 90 points, respectively, and the visual analog scale pain score was at 1 point. The range of motion of the ankle joint-- injured° (uninjured°)was checked presenting an ankle dorsiflexion of 15° (20°), an ankle plantar flexion of 40° (40°), a varus of 20° (20°), and a valgus of 10° (10°), showing almost no limitations.

## 3. Discussion

Traumatic distal tibiofibular syndesmosis injuries commonly occur during contact sports. Syndesmotic injuries that are associated with ankle rotation account for approximately 10% of all ankle fractures, >20% of which are treated surgically [3,4]. A retrospective study found that the proportion of syndesmotic injuries that were sustained by athletes that could be classified as acute sprains was approximately 20% [23,24]. Missed or improperly treated syndesmosis injuries can result in unnecessary pain or functional impairment, which may ultimately progress to arthritis [25,26]. Achieving and maintaining an anatomical reduction is important for good long-term, complication-free outcomes in cases of syndesmotic injury [25].

The treatment methods for distal syndesmosis injuries are highly controversial [3,9,27]. The traditional fixation method for an unstable syndesmosis is transsyndesmotic screw fixation. Although the number of screws, the fixation period, and the removal time are debatable, this traditional fixation is still the most widely used technique. However, its disadvantages include screw breakage, malreduction, synostosis, the need for screw removal (and diastasis thereafter), delayed weight-bearing, and disuse osteoporosis [28,29,30]. Good outcomes of suture-button fixation have been reported by studies that applied this technique to overcome the drawbacks of the traditional fixation [8,27,29,31]. However, the potential complications of suture-button fixation include soft tissue complications, infections, osteolysis, and heterotopic ossification [32,33,34]. In a biomechanical study, suture-button fixation alone did not provide an adequate rotational stability [21,35]. Forsythe et al. reported that FiberWire-button (Arthrex) fixation was less effective for maintaining syndesmotic reduction in the immediate postoperative period, relative to a metallic screw [36]. Moreover, Teramoto et al. reported that neither single- nor double-suture-button fixation stabilized the syndesmosis in cases of inversion and external rotation, although the former was sufficient for physiologic stability [37].

Several studies have reported good results from using suture tape in conjunction with suture-button fixation for an AITFL augmentation [21,35]. Nonabsorbable suture tape that is designed for the treatment of ankle lateral instability has been widely applied, while the InternalBrace (Arthrex) was developed in 2012. This device uses SwiveLock screws for a knotless aperture fixation, and FiberTape (Arthrex) fixed to each ligament enhances the repair and augmentation.

Nelson proposed an open anatomic repair for AITFL injuries, and reported that this technique can restore the ankle’s mortise stability and facilitate bone repair, in order to promote an early return to functional exercises and activities [38]. Moreover, there is no requirement for a syndesmotic screw fixation. Lee et al. introduced a repair technique for the AITFL by using suture tape under arthroscopic guidance [39]. Although their approach has a basic concept similar to that of Nelson, it also has distinct advantages in terms of weight-bearing and rehabilitation in the early stage after surgery, a lack of any requirements for screw removal, and no functional limitations [38,39]. Kwon et al. reported that the use of the InternalBrace for AITFL injuries was an effective and safe adjunctive strategy for addressing syndesmotic instability [19]. Lee et al. reported that open anterior syndesmotic repair using suture tape provided a torsional strength that was similar to screw fixation in cases of ankle syndesmotic injury, and suggested that it could serve as an alternative treatment option [21].

The suture tape techniques described above have a notable limitation: they can only be performed when the PITFL is intact. In a cadaver model, Regauer et al. introduced a minimally invasive anterior and posterior augmentation technique using the InternalBrace device [22]. When using such techniques in actual patients, an initial examination should be performed to determine whether the patient is a suitable candidate. If a PITFL rupture is confirmed by an ankle axial CT, an MRI, and arthroscopy, and if a reduction is also deemed to be required, the AITFL and PITFL augmentation can be performed using InternalBrace. To confirm a successful surgical outcome when using the InternalBrace fixation, the degree of syndesmosis reduction should be assessed by an axial CT immediately, through a comparison with the uninjured side.

## 4. Conclusions

As a treatment for unstable syndesmosis injury, a ligament augmentation using suture tape provides satisfactory clinical outcomes and can be considered to be a useful and reliable method for anatomical restoration and rapid rehabilitation. However, cadaveric biomechanical studies are needed for validation.

## Data Availability

Data sharing is not applicable to this article as no datasets were generated or analyzed during the current study.

## Abbreviations

AITFL	anterior inferior tibiofibular ligament
IOL	interosseous ligament
TL	transverse ligament
PITFL	posterior inferior tibiofibular ligament
CT	computed tomography
MRI	magnetic resonance imaging.
